# The complete mitochondrial genome and phylogenetic position of the half-fin anchovy, *Setipinna tenuifilis* (Valenciennes, 1848)

**DOI:** 10.1080/23802359.2018.1462127

**Published:** 2018-04-23

**Authors:** Huimin Fan, Qiqun Cheng, Kaisong Peng

**Affiliations:** a Key Laboratory of Oceanic and Polar Fisheries, Ministry of Agriculture, East China Sea Fisheries Research Institute, Chinese Academy of Fishery Sciences, Shanghai, China;; b Laboratory of Aquatic Health and Public Health, College of Animal Science and Technology, Anhui Agricultural University, Anhui, China

**Keywords:** Half-fin anchovy, *Setipinna tenuifilis*, mitochondrial genome, molecular taxonomy, phylogenetic relationship

## Abstract

Half-fin anchovy (*Setipinna tenuifilis*) is one of the most important economic fishes around the world. In the present study, we determined the complete mitochondrial DNA sequence and organization of *S. tenuifilis*. The entire mitochondrial genome is a circular-molecule of 16,215 bp in length, which encodes 37 genes in all. These genes comprise 13 protein-coding genes (ATP6 and 8, COI–III, Cytb, ND1-6, and 4L), 22 transfer RNA genes (tRNAs), and two ribosomal RNA genes (12S and 16S rRNAs), with gene arrangement and content basically identical to those of other species of Engraulidae. The result of phylogenetic analysis strongly supported that *S. tenuifilis* was first clustered together with *Setipinna melanochir* and formed a monophyly in the genus *Coilia*, and then they constituted a sister-group relationship with two genus *Engraulis*, and *Stolephorus*. It concluded that the *S. tenuifilis* should be classified into the genus *Setipinna*. The present study also revealed the phylogenetic relationship of this genus at molecular levels. The complete mitochondrial genome sequence of *S. tenuifilis* can provide basic information for the studies on molecular taxonomy and phylogeny of teleost fishes.

Half-fin anchovy (*Setipinna tenuifilis*), one of the most important economic fishes, is a highly migratory species distributed in the Indian Ocean and Western Pacific Ocean. Till now, we know little about mitogenome of *S. tenuifilis*. Thus, we determined the complete mitogenome sequence and organization of *S. tenuifilis* here.

*Setipinna tenuifilis* specimens were collected from Shanghai, China and deposited in East China Sea Fisheries Research Institute. The complete mitogenome sequence of *S. tenuifilis* was determined by polymerase chain reaction amplification and sequencing, using 13 pairs of primers which were designed based on mitogenome sequence of *Setipinna melanochir* (GenBank number AP011565.1), a closely related species of *S. tenuifilis*. The complete mitogenome of *S. tenuifilis* was deposited in GenBank database with accession number MH037012. Muscle tissues were persevered in 95% ethanol for DNA extraction. Total genomic DNA was extracted from muscle tissue by standard phenol–chloroform procedure (Sambrook et al. 1989).

The complete mitogenome of *S. tenuifilis* was determined to be 16,215 bp, comprising 37 coding and two non-coding regions. The 37 coding regions include 13 protein-coding genes (ATP6 and 8, COI–III, Cytb, ND1-6, and 4L), 22 transfer RNA genes (tRNAs), and two ribosomal RNA genes (12S and 16S rRNAs), and two non-coding regions consist of light-strand replication origin (O_L_) and control region (CR). Except for one protein-coding gene ND6 and eight tRNAs (*tRNA^Gln^*, *tRNA^Ala^*, *tRNA^Asn^*, *tRNA^Cys^*, *tRNA^Tyr^*, *tRNA^Ser^*, *tRNA^Glu^*, and *tRNA^Pro^*), all other genes are encoded on the heavy strand, which are in accordance with other teleost mitogenomes (Zhu et al. 2013).

The overall nucleotide base composition of *S. tenuifilis* mitogenome is as follows: A, 30.90%; G, 15.70%; T, 25.00%; and C, 28.40%, respectively, showing an obvious anti-G bias as appeared in other teleost species (Jondeung et al. 2007).

The CR of *S. tenuifilis* is 1254 bp in length, with *tRNA^Pro^* and *tRNA^Phe^* at its two ends. The O_L_ is 29 bp long, locating in a cluster of the *tRNA^Trp^-tRNA^Ala^-tRNA^Asn^-tRNA^Cys^-tRNA^Tyr^* region (WANCY region, Table 1).

**Table 1.** Characteristics of the mitochondrial genome of *Setipinna tenuifilis*.

Locus	Abbreviation	Position		Size nucleotide (bp)	Codon		Amino acid (AA)	Anti-codon	Intergenic nucleotide	Strand
		From	To		Start	Stop				
*tRNA^*Phe*^*	F	1	69	69				GAA	0	H
*12S rRNA*	12S	70	1020	951					0	H
*tRNA^*Val*^*	V	1021	1092	72				TAC	0	H
*16S rRNA*	16S	1093	2779	1687					14	H
*tRNA^*Leu*^*	L	2794	2868	75				TAA	0	H
*ND 1*	nd1	2869	3807	939	ATG	CTA	313		42	H
*tRNA^*Ile*^*	I	3850	3921	72				GAT	−1	H
*tRNA^*Gln*^*	Q	3921	3991	71				TTG	−1	L
*tRNA^*Met*^*	M	3991	4059	69				CAT	0	H
*ND 2*	nd2	4060	5067	1008	ATG	ATT	336		7	H
*tRNA^*Trp*^*	W	5075	5146	72				TCA	2	H
*tRNA^*Ala*^*	A	5149	5217	69				TGC	1	L
*tRNA^*Asn*^*	N	5219	5291	73				GTT	0	L
Rep origin	O_L_	5292	5320	29					0	
*tRNA^*Cys*^*	C	5321	5387	67				GCA	0	L
*tRNA^*Tyr*^*	Y	5388	5458	71				GTA	7	L
*COX I*	cox1	5466	6989	1524	ATC	ATG	508		5	H
*tRNA^*Ser*^*	S	6995	7065	71				TGA	5	L
*tRNA^*Adp*^*	D	7071	7139	69				GTC	11	H
*COX II*	cox2	7151	7813	663	ATG	CAA	221		30	H
*tRNA^*Lys*^*	K	7844	7916	73				TTT	1	H
*ATP 8*	atp8	7918	8082	165	ATG	CAT	55		−7	H
*ATP 6*	atp6	8076	8756	681	ATG	CTG	227		2	H
*COX III*	cox3	8759	9541	783	ATG	ACT	261		2	H
*tRNA^*Gly*^*	G	9544	9615	72				TCC	0	H
*ND 3*	nd3	9616	9963	348	ATG	AAG	116		1	H
*tRNA^*Arg*^*	R	9965	10,033	69				TCG	0	H
*ND4L*	nd4L	10,034	10,327	294	ATG	CGT	98		−4	H
*ND 4*	nd4	10,324	11,694	1371	ATG	CGG	457		10	H
*tRNA^*His*^*	H	11,705	11,772	68				GTG	1	H
*tRNA^*Ser*^*	S	11,774	11,840	67				GCT	0	H
*tRNA^*Leu*^*	L	11,841	11,912	72				TAG	0	H
*ND 5*	nd5	11,913	13,039	1127	ATG	ATC	375		5	H
*ND 6*	nd6	13,045	13,563	519	AAC	TAC	173		0	L
*tRNA^*Glu*^*	E	13,564	13,632	69				TTC	4	L
*Cyt b*	cyt b	13,637	14,767	1131	ATG	TTC	377		55	H
*tRNA^*Thr*^*	T	14,823	14,891	69				TGT	−1	H
*tRNA^*Pro*^*	P	14,891	14,961	71				TGG	0	L
Control region	CR	14,962	16,215	1254					0	

All protein-coding genes in *S. tenuifilis* have a methionine (ATG) start codon except for *COI* and *ND 6*, which starts with ATC and AAC. The 13 protein-coding genes (*ND1*, *ND2*, *COI*, *COII*, *ATP8*, *ATP6*, *COIII*, *ND3*, *ND4L*, *ND4*, *ND5*, *ND6*, and *Cytb*) are ended with CTA, ATT, ATG, CAA, CAT, CTG, ACT, AAG, CGT, CGG, ATC, TAC, and TTC (Table 1).

There are some overlaps existing in *S. tenuifilis* mitogenome. For 13 protein-coding genes, there are two overlaps detected in *ATP8-ATP6* and *ND4-ND4L*. Among 22 tRNAs, three overlaps are found in *tRNA^Ile^-tRNA^Gln^*, *tRNA^Gln^-tRNA^Met^*, and *tRNA^Thr^-tRNA^Pro^* (Table 1).

Phylogenetic analysis was performed by MEGA 6.06 (Tamura et al. 2013) based on complete mitogenome sequence of *S. tenuifilis* and those of 13 closely related species belonging to four genus *Engraulis*, *Stolephorus*, *Setipinna*, and *Coilia.* The neighbour-joining tree (Figure 1) showed that *S. tenuifilis* first clustered together with *Setipinna melanochir* and formed a monophyly in the genus *Coilia*, and then they constituted a sister-group relationship with other two genus. This result strongly supported that *S. tenuifilis* should be classified into the genus *Setipinna*. This study also revealed the phylogenetic relationship of the genus *Coilia* at molecular levels.

**Figure 1.**
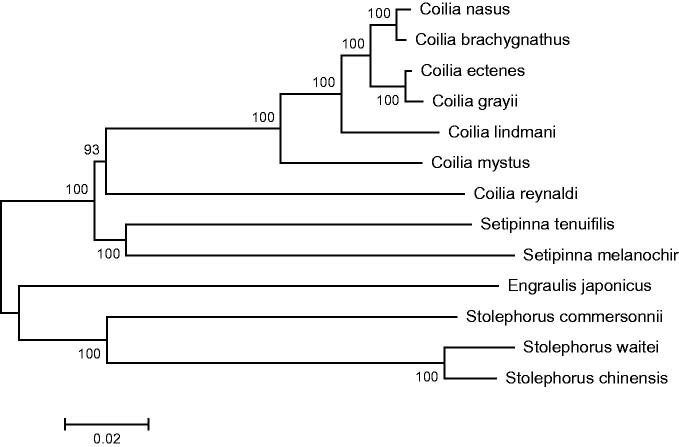
Phylogenetic position of the half-fin anchovy.
